# Magnetic Mitohormesis as a Potential Non-Invasive Restorative Therapy for X-Linked Muscular Dystrophies

**DOI:** 10.3390/ijms27177700

**Published:** 2026-08-28

**Authors:** Jan Nikolas Iversen, Alfredo Franco-Obregón

**Affiliations:** 1 Department of Surgery, Yong Loo Lin School of Medicine, National University of Singapore, Singapore 119228, Singapore; iversen@nus.edu.sg; 2 Institute of Health Technology and Innovation (iHealthtech), National University of Singapore, Singapore 117599, Singapore; 3 BICEPS Lab (Biolonic Currents Electromagnetic Pulsing Systems), National University of Singapore, Singapore 117599, Singapore; 4 NUS Centre for Cancer Research (N2CR), Yong Loo Lin School of Medicine, National University of Singapore, Singapore 117597, Singapore; 5 Competence Center for Applied Biotechnology and Molecular Medicine, University of Zürich, 8057 Zürich, Switzerland

**Keywords:** X-linked muscular dystrophy, Duchenne muscular dystrophy, TRPC1, mechanotransduction, calcineurin signalling, PGC-1α, utrophin, magnetic mitohormesis

## Abstract

Duchenne and Becker X-linked muscular dystrophies are no longer viewed as disorders arising solely from passive sarcolemmal fragility but as diseases that progress because of disruption of cellular mechanotransduction. Evidence is accumulating that the gating of TRPC1 and TRPC3 mechanosensitive channels is altered in the absence of dystrophin, resulting in a breakdown of sarcoplasmic calcium homeostasis and preferential loss of type II glycolytic muscle fibres, while type I oxidative fibres are spared. TRPC1-mediated Ca^2+^ influx is known to activate the calcineurin–NFAT pathway, upregulating PGC-1α transcriptional activity to promote mitochondriogenesis, antioxidant defences, and the oxidative muscle phenotype. Calcineurin signalling also activates a compensatory pathway in dystrophinless muscle by inducing the expression of utrophin, a dystrophin autosomal homologue, capable of substituting for dystrophin at the muscle surface. This perspective explores the possibility that non-mechanical biophysical stimuli can be used to restore calcineurin signalling by non-invasively activating TRPC1. Pulsed electromagnetic field (PEMF) exposure has been shown to stimulate TRPC1-mediated Ca^2+^ entry and calcineurin-dependent signalling in skeletal muscle and may serve as a gentle method to induce utrophin expression in X-linked muscular dystrophies. By activating calcineurin compensatory mechanisms in these disorders, PEMF-based paradigms warrant future investigation as mechanistically grounded adjuvants to conventional therapies.

## 1. Muscular Dystrophy as a Disease of Disrupted Mechanotransduction

Muscular dystrophies are a group of genetic diseases characterised by progressive muscle weakness and atrophy [1]. More than 30 different types of muscular dystrophy are known, which all have in common difficulties in walking and in maintaining balance, leading to heightened risks of falling and injury [2]. Duchenne muscular dystrophy (DMD) is the most prevalent form of muscular dystrophy, accounting for roughly one-third to half of all muscular dystrophy cases globally [3], almost entirely in males [4]. The onset of symptoms in DMD occurs in early childhood and includes muscle weakness and degeneration, particularly of the leg, pelvis and core musculature, resulting in premature death due to cardiac or respiratory failure [5]. Becker muscular dystrophy (BMD) is similar in aetiology to DMD, but with milder symptomology, appearing in adolescence with slower progression [6]. Both DMD and BMD arise from mutations of the dystrophin gene located on the X chromosome, resulting either in its complete absence or in the elaboration of truncated versions of the protein, respectively.

Dystrophin is a key component of the dystrophin–glycoprotein complex (DGC). The DGC is a large multi-protein complex that serves as a stabilising structural intermediate between the extracellular matrix and the intracellular cytoskeleton of muscle [7,8]. Externally, the DGC anchors the muscle surface to the extracellular matrix. Internally, dystrophin mediates the interaction of the DGC with the muscle cytoskeleton via its N-terminal domain that binds to costameric actin originating from the Z-disc of the sarcomere [9]. In this manner, the DGC acts as a molecular spring to absorb mechanical stresses arising during muscle contraction (shortening of the sarcomere), effectively transferring internal mechanical stresses to the muscle’s exterior. A functioning DGC helps prevent muscle membrane damage during normal physical activity. Consequently, the loss of dystrophin disrupts an essential structural scaffolding complex of muscle and alters the proper function of DGC-associated molecular signalling complexes, such as ion channels, that are required to translate external and internal mechanical forces into enzymatic responses necessary for adaptation and survival. Most commonly, stretch or mechanosensitive channels have been implicated in the disruption [10,11]. Nonetheless, the activity of voltage-gated and intracellular channel classes has also been reported to be altered [12]. Fundamentally, therefore, DMD and BMD are structural disorders linked to sarcolemma fragility and consequent contraction-mediated muscle necrosis [13].

The membrane fragility interpretation of X-linked muscular dystrophies, however, fails to account for all aspects of disease progression, including mitochondrial dysfunction and oxidative stress. A growing body of evidence now indicates that the loss of dystrophin disrupts cellular mechanotransduction, by altering how mechanical forces are converted into cellular signalling cascades [14]. Muscular mechanotransduction is encoded by Ca^2+^ increments of catalytic capacity. Specifically, Ca^2+^ acts as a cofactor that regulates the activity of enzymes involved in muscle development and adaptation. One common hypothesis is that dystrophin, via its innate cytoskeletal interactions, mediates the transfer of mechanical information to calcium-permeable stretch-activated channels. In the absence of dystrophin, however, the DGC would be unable to correctly convey mechanical information to stretch-activated channels, compromising their ability to transduce mechanical signals into appropriate cellular responses and resulting in maladaptive Ca^2+^ entry and impaired adaptive calcium signalling [15]. A corollary to this hypothesis asserts that the absence of dystrophin at the DGC shifts the gating threshold of stretch-activated channels to lower levels [16,17], resulting in exaggerated resting Ca^2+^ entry at resting membrane tensions. What then ensues is a breakdown in intracellular Ca^2+^ homeostasis and a state of generalised degenerative calcium-dependent proteolysis. What is more difficult to ascertain is the degree to which mechanically induced membrane damage, beyond the loss of dystrophin, contributes to the maladaptive gating of stretch-activated channels. The downstream consequences of stretch-activated channel-mediated Ca^2+^ overload include chronic inflammation, mitochondrial dysfunction, impaired regenerative capability and satellite cell exhaustion, exacerbating the condition [18]. The altered activities of mechanosensitive TRP channels, such as TRPC1 and TRPC3, are frequently implicated in this process [19], whereby TRPC1 has been shown to directly interact with the DGC complex [20,21]. This perspective will focus on the TRPC1 and TRPC3 channels due to the weight of available evidence implicating their involvement in the dystrophic condition.

Current therapeutic strategies, including corticosteroids, exon skipping and gene replacement, primarily target the symptoms or the upstream genetic defects, but may not restore disrupted mechanotransductive signalling networks [22]. In particular, gene delivery is hampered by the extremely large coding sequence of the dystrophin gene (>11–14 kb), restricting the method used to “micro-dystrophin” genes, which will inevitably sacrifice essential functional domains and may not fully restore function to the dystrophin signalling complex [23,24]. As current adeno-associated viral (AAV) vectors have a packaging capacity of approximately 4.7 kb [25], several regions of the full-length dystrophin protein involved in stabilising the sarcolemma and coordinating interactions with components of the DGC are necessarily omitted, such that the use of truncated micro-dystrophin constructs may only restore part of the structural and signalling functions of native dystrophin [23,24]. Gene delivery may also incite host immune and inflammatory responses, as well as face the danger that the replaced gene may be eventually lost due to the rapid turnover of diseased muscle. Moreover, the delivery of a replacement gene may be limited due to the incomplete penetrance of the vector to critical deeply embedded muscles. A therapeutic gap hence exists for interventions that can thoroughly and repeatedly, if necessary, reinstate functional calcium signalling, rather than relying on burdensome therapies to partially restore dystrophin deficiency or reduce muscle wastage without addressing the underlying cause of muscle deterioration.

## 2. Mechanosensitive Calcium Dysregulation in Dystrophic Muscle

Aberrant stretch-activated channel activity has long been implicated in pathophysiology of DMD with much of the literature focusing on its role in supporting pathological Ca^2+^ overload [10,11]. Early studies showed that muscle fibres lacking dystrophin were more susceptible to stretch-induced membrane damage, resulting in Ca^2+^ leakage into the sarcoplasm. This finding initially supported the interpretation that normal levels of physical activity were sufficient in muscle lacking dystrophin to produce membrane tears that led to the non-discriminate leakage of Ca^2+^ into the sarcoplasm [10,26]. However, extracellular Ca^2+^ entry as well as muscle pathology were later shown to be prevented by the administration of pharmacological antagonists of stretch-activated channels, indicating that Ca^2+^ entry was cation channel-mediated [27,28,29,30,31,32]. More recently, the mechanosensitive cation channel class most closely implicated as the source of maladaptive Ca^2+^ influx driving dystrophic muscle fibre degeneration has been identified as the mechanosensitive TRPC channels, particularly, the TRPC1 and TRPC3 channel pair [26,33,34,35,36,37].

Under normal conditions TRPC channel-mediated Ca^2+^ signalling is essential for skeletal muscle adaptation, repair and fibre-type specification, but, when deregulated, can become pathological in nature [38,39]. Along with other TRP channel classes [40], TRPC1 and TRPC3 are known mediators of skeletal muscle mechanotransduction [41,42]. TRPC1-mediated Ca^2+^ entry activates transcriptional programmes governing early myogenesis towards the oxidative phenotype in response to mechanical loading [27,43,44], consistent with observations that TRPC1 expression is highest in slow oxidative muscle fibres [45] and decreases with mechanical unloading in parallel with a loss of oxidative phenotype [27,46]. TRPC3, on the other hand, typically plays a predominant role in excitation–contraction coupling by supporting sarcoplasmic reticulum Ca^2+^ release via the ryanodine receptor (RyR1) [47]. Under normal conditions, TRPC3 is thus predominantly found at the transverse-tubules (T-tubules) [48], but in DMD, TRPC3 associates with NADPH oxidase (Nox2) at the muscle surface [49,50]. By contrast, TRPC1 is normally found at the surface of skeletal muscle where it interacts with dystrophin via another DGC-associated protein, known as α_1_-syntrophin. The indirect association of dystrophin with TRPC1 thus positions it as an inherent part of the muscular (skeletal, cardiac and vascular) mechanotransductive machinery that is normally responsible for translating mechanical input into adaptive calcium signalling [8,51]. Consequently, the disruption of the DGC, caused by the absence of dystrophin, compromises both membrane stability as well as TRPC1-dependent mechanotransduction [8]. How the absence of dystrophin ultimately changes the interactions between TRPC1 and TRPC3 [52] and alters their function is an active area of ongoing research.

TRPC1 and TRPC3 channels appear to be activated asynchronously across the sarcolemma in response to different mechanical and biochemical stimuli, consistent with their distinct and opposing roles in muscle [8,53]. While both TRPC1 and TRPC3 are upregulated in dystrophic muscle and may individually, or as a heteromultimer, contribute to the observed alterations in calcium homeostasis, it is TRPC3 that has been identified as the key early contributor to maladaptive calcium overload and downstream activation of proteolytic and physiological pathways that result in progressive muscle fibre degeneration [33,34]. Under pathological conditions, the C-terminus of TRPC3 binds to the catalytic subunit of the NADPH oxidase enzyme complex, Nox2, at the muscle surface. The formation of a stable TRPC3–Nox2 complex strongly drives the formation of reactive oxygen species (ROS), which further augments TRPC3 channel activity. This detrimental, positively reinforcing interaction ultimately produces an overwhelming level of oxidative stress that leads to muscle degeneration. In the *mdx* mouse model of X-linked muscular dystrophy, suppression of the TRPC3-Nox2 interaction ameliorated muscle atrophy and improved strength [50]. Moreover, inhibition of TRPC3 was sufficient to abolish exaggerated Ca^2+^ entry into dystrophic skeletal muscle fibres, restoring sarcolemmal Ca^2+^ influx to normal levels and preventing intracellular calcium overload [35]. Similar benefits to TRPC3 antagonism were also observed in vascular smooth muscle cells from the *mdx* mouse [54].

To a large degree, TRPC1 may be implicated in the dystrophic pathology merely by association with TRPC3. Recent evidence indicates that their specific contributions and timelines of expression in the disease differ. TRPC3 expression and activity are upregulated very early on in the disease, whereas TRPC1 upregulation occurs much later [35] when an observed muscle fibre transition to the oxidative phenotype is apparent [55]. Moreover, specific inhibition of TRPC3 abolishes the differences in calcium permeability between healthy and dystrophic muscles [35]. TRPC1-mediated Ca^2+^ entry activates transcriptional programmes governing muscular oxidative phenotypic remodelling [34,43,44,45] and mitochondrial survival adaptations leading to heightened resistance against oxidative stress [56,57,58], which would confer a survival advantage in DMD. Ironically, elevated TRPC1-mediated Ca^2+^ entry in the dystrophic condition may be the reason for the glycolytic to oxidative shift naturally observed in X-linked muscular dystrophies [27,45].

## 3. TRPC1-Dependent Calcineurin Signalling Promotes Oxidative Muscle Development

In DMD, the degree of muscle degeneration depends on the relative proportion of oxidative to glycolytic fibres within a muscle [59]. One of the characteristic features of DMD is a preferential vulnerability of type II fast-twitch glycolytic muscle fibres [55,60]. Fast glycolytic fibres are affected earlier and more severely than slow oxidative muscle fibres [39]. Moreover, in an adaptive stress response to the absence of dystrophin, type II fast-twitch glycolytic muscle fibres transition over to a slower, more oxidative phenotype [55]. Oxidative muscle fibres also possess greater mitochondrial density and resistance to oxidative stress than glycolytic fibres [61], conferring upon them an intrinsic survival advantage in DMD [55,62].

Oxidative muscles also exhibit the highest expression levels of TRPC1 [45], where it promotes oxidative (type I) muscle development [27,43,46]. By contrast, the expression of TRPC3 is induced by mechanical forces in both fast glycolytic (type II) and slow oxidative (type I) muscle [63], and its expression is often inversely correlated to that of TRPC1 [64]. Both TRPC1 (TRPC1/4/5 subfamily) and TRPC3 (TRPC3/6/7 subfamily) are Ca^2+^-permeable and have reported mechanosensitivity, but with disparate developmental principles [40]. TRPC1 acts as a general regulator of other TRPC channel members [42] and is responsive to diverse electromagnetic biophysical stimuli that it confers to TRPC heteromultimers [57,65]. In contrast, TRPC3 can act as a standalone homomultimer that responds to phosphatidylinositol-derived diacylglycerol produced by the membrane-associated phospholipase C signalling cascade activated by surface membrane mechanosensitive G-protein coupled receptors [66]. TRPC3 is hence indirectly mechanosensitive [67], an attribute that may be reinforced by its association with TRPC1 [42]. TRPC3 is commonly upregulated in pathological states, causing calcium overload and oxidative stress [50], whereas TRPC1-mediated Ca^2+^ entry is adaptive in nature, in the mitohormetic sense [46,68,69]. Most critically, TRPC1 acts to oppose TRPC3-mediated Ca^2+^ entry [46,68,69]. A manner to activate TRPC1, with minimal mechanical stress and a high control over stimulation intensity, may thus provide a means to revert the deleterious consequences associated with TRPC3 hyperactivity in DMD.

Calcineurin is a calcium/calmodulin-activated serine-threonine phosphatase that translates mechanical stimulation into calcium-coded transcriptional responses [70]. In skeletal muscle, TRPC-mediated Ca^2+^ entry stimulates the calcineurin-dependent dephosphorylation of the nuclear factor of activated T-cells (NFAT) that regulates transcriptional responses governing muscle metabolic phenotype determination along glycolytic or oxidative lines. The NFATc1 isoform is selectively activated by sustained intracellular Ca^2+^ signalling, whereas transient and high-amplitude Ca^2+^ elevations preferentially activate NFATc2 [71]. Once NFATc1 is dephosphorylated by calcineurin, its translocation into the nucleus regulates transcriptional programmes associated with the oxidative muscle phenotype, such as the slow isoforms of the myosin heavy chain, troponin, MyoD (upstream of myogenin) and myoglobin [5,56,58,71,72]. In summary, NFATc1 drives the fast (glycolytic)-to-slow (oxidative) muscle fibre type transition typically associated with exercise [73] and NFATc2 is more dedicated to promoting muscle fibre growth [74]. Finally, selective activation of TRPC1 by biophysical means was shown to activate NFATc1 as well as promote oxidative muscle determination [43,56]. The TRPC1 signalling axis hence may remain therapeutically accessible via biophysical means to promote the expression of the protective oxidative phenotype in dystrophic muscle [27,46].

Calcineurin also directly dephosphorylates the myocyte enhancer factor-2 (MEF2) transcription factor. In its hypophosphorylated state, MEF2 is able to bind A/T-rich consensus promoter DNA to activate transcription. MEF2 and NFAT also physically interact within the nucleus to coordinate transcriptional activity related to oxidative metabolism, mitochondrial biogenesis, and slow-twitch myofibre specialisation [75]. By contrast, class II histone deacetylases (HDACs) repress MEF2 activity to restrict the basal transcription of genes associated with the oxidative muscle phenotype. Sustained calcineurin- and calcium-calmodulin-dependent phosphatase/kinase cascades phosphorylate HDACs, causing them to dissociate from MEF2 and promote their export from the nucleus, permitting the transcription of genes involved in mitochondrial adaptation and oxidative metabolism [56,58,72]. The simultaneous activation of NFAT together with the release of MEF2 from HDAC-mediated repression allows these transcription factors to cooperate at specific promoters regulating the oxidative muscle programme. Through the coordinated mediation of NFAT- and MEF2-dependent gene programmes, calcineurin functions as a central integrator for coupling mechanosensitive calcium signalling to skeletal muscle remodelling.

The major target of the NFATc1–MEF2C complex is the upstream promoter region of the proliferator-activated receptor gamma coactivator-1 alpha (PGC-1α) gene, the master regulator of mitochondrial biogenesis and oxidative metabolism [56,58,72,76,77,78]. Increased PGC-1α expression, in turn, promotes muscle adaptation by driving the transcription of genes regulating mitochondrial content, fatty acid utilisation, aerobic respiration and fatigue-resistant muscle performance, hallmarks of oxidative muscle. Importantly, PGC-1α can further reinforce oxidative transcriptional programmes through autoregulatory mechanisms that sustain its expression, revealing the existence of a self-amplifying network that fortifies oxidative muscle expression [79]. Therefore, calcineurin-NFATc1 activation resulting from TRPC1-mediated Ca^2+^ entry, by stimulating PGC-1α transcriptional activity, promotes adaptive oxidative muscle development such as typically observed with exercise training [73].

## 4. Calcineurin as a Recovery Factor in X-Linked Muscular Dystrophy

Chakkalakal and colleagues demonstrated the functional importance of calcineurin signalling in dystrophic muscle. Crossing *mdx* mice with transgenic mice expressing a constitutively active form of calcineurin (CnA*), they demonstrated ameliorated muscle pathology and a shift in muscle fibres towards the slower oxidative muscle phenotype in association with enhanced nuclear localisation of NFAT [80]. Muscles from the *mdx*/CnA* mice exhibited an approximately two-fold increase in utrophin expression, together with the restoration of multiple components of the DGC at the sarcolemma. The structural restoration of the DGC also reduced membrane fragility as evidenced by the greater exclusion of Evans blue dye and serum IgM and albumin into the sarcoplasm. The infiltration of muscle by inflammatory immune cells and histopathological indicators of muscle degeneration were also reduced in the crossed mice. These findings collectively illustrate the broad restorative capacity of calcineurin signalling in the dystrophic scenario [80].

**Figure 1:**
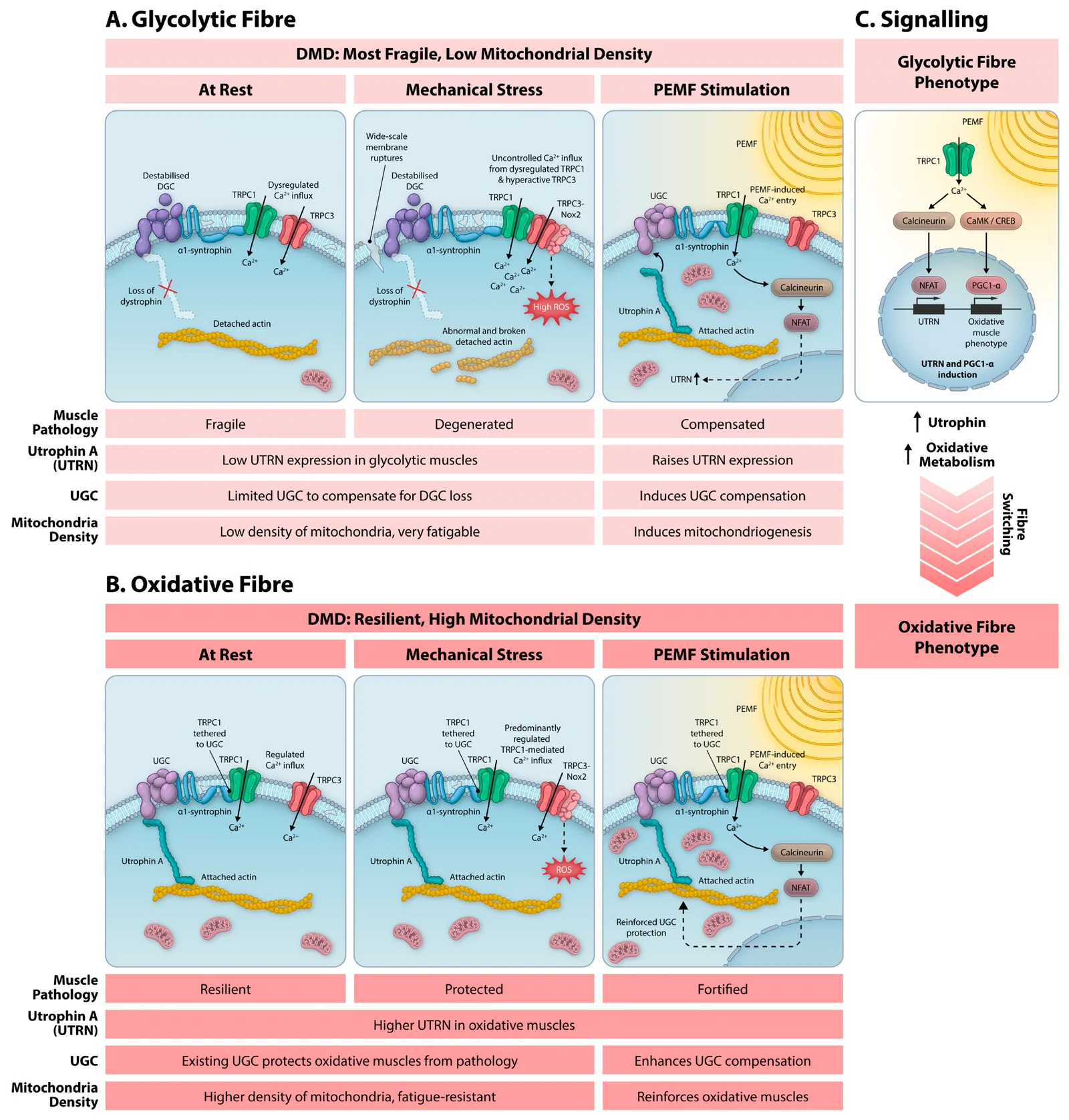
**TRPC1-mediated calcineurin activation and utrophin induction in dystrophin-deficient muscle.** *(Interactions of the DGC and UGC with extracellular laminin via α-dystroglycan are omitted for clarity).* (**A**) **Glycolytic muscle fibres:** Vulnerable phenotype characterised by low mitochondrial density and basal utrophin expression. Loss of dystrophin destabilises the DGC and disrupts TRPC1 mechanotransduction. Under mechanical stress, sarcolemmal rupture and dysregulated TRPC1/3 activity cause excessive Ca^2+^ influx, high ROS, and fibre degeneration. PEMF stimulation may trigger non-mechanical TRPC1-mediated Ca^2+^ entry, activating calcineurin–NFAT signalling to upregulate utrophin and induce UGC compensation. (**B**) **Oxidative muscle fibres:** Resilient phenotype characterised by high mitochondrial density and basal utrophin expression. Preserved membrane stability maintains UGC–TRPC1 tethering, limiting pathological Ca^2+^ entry and ROS during mechanical stress. PEMF enhances this protective profile by reinforcing UGC stability and oxidative capacity. (**C**) **Signalling mechanism and phenotypic remodelling:** PEMF-induced TRPC1-mediated Ca^2+^ influx activates calcineurin–NFAT (driving UTRN expression) and CaMK/CREB–PGC-1α signalling (promoting oxidative metabolism). The central downward arrow indicates directional remodelling from fragile glycolytic fibres (light pink) toward a resilient oxidative phenotype (dark pink) following PEMF stimulation. Colour gradients visually illustrate phenotypic muscle fibre states. **Abbreviations: CaMK:** Ca^2+^/Calmodulin-dependent protein kinase; **CREB:** cAMP response element-binding protein; **DGC:** Dystrophin–glycoprotein complex; **NFAT:** Nuclear factor of activated T-cells; **Nox2:** NADPH oxidase 2; **PEMF:** Pulsed electromagnetic field; **PGC-1α:** Peroxisome proliferator-activated receptor gamma coactivator 1-alpha; **ROS:** Reactive oxygen species; **TRPC1/3:** Transient receptor potential channel canonical 1/3; **UGC:** Utrophin–glycoprotein complex; and **UTRN:** Utrophin.

The utrophin proteins (395 kDa) are autosomal homologues of dystrophin (427 kDa) that are differentially expressed across diverse tissue types [81,82,83]. Specifically, utrophin A isoform is enriched in oxidative muscle fibres where its expression correlates with oxidative capacity [81,84]. In normal skeletal muscle, utrophin is expressed predominantly at the neuromuscular junction, whereas dystrophin is typically expressed at the muscle surface in the context of the DGC [81]. In DMD, however, utrophin expression is upregulated and redirected to the sarcolemma. Stimulation of calcineurin signalling is responsible for the upregulation of muscle utrophin in DMD, which then compensates for the absence of dystrophin at the DGC [80]. The compensatory induction of utrophin expression by calcineurin activation provides a mechanistic basis for understanding the preferential survival of oxidative muscle in DMD (Figure 1). Developing methods to activate calcineurin without requiring mechanical stress may improve patient prognosis in DMD.

In surviving dystrophic muscle fibres, utrophin assembles a compensatory utrophin–glycoprotein complex (UGC) at the sarcolemma to replace the damaged DGCs (lacking dystrophin) [85,86]. Although the UGC re-establishes key interactions of the muscle membrane with the extracellular matrix and cytoskeleton, it does not fully restore all native DGC structural and signalling functions. Notably, the UGC lacks the ability to bind and anchor neuronal nitric oxide synthase (nNOS), though it still improves sarcolemma stability and reduces susceptibility to contraction-induced injury [86,87]. TRPC1 also associates with the UGC via its interaction with α_1_-syntrophin, recreating an analogous interaction of TRPC1 with the DGC via α_1_-syntrophin [20,21]. Utrophin reverts aberrant TRPC1 stretch-activated channel gating in isolated muscle fibres from the *mdx* mouse to a more normal mode of activation [88,89]. The partial restoration of muscle cytoarchitecture [90] and DGC “function” [88,89] by the replacement of dystrophin (DGC) by utrophin (UGC) in the *mdx* mouse thus provides some mechanistic basis for understanding the greater resilience of oxidative muscle fibres when dystrophin function is compromised.

Calcineurin’s regulation of utrophin expression is multidimensional [84]. First, calcineurin activation stimulates the transcriptional activity of the utrophin A promoter mediated by NFAT, whereas calcineurin inhibition markedly reduces utrophin A mRNA levels [84]. Calcineurin signalling also enhances utrophin A mRNA stability through an AU-rich element within the 3’ untranslated region, providing an additional post-transcriptional mechanism for maintaining elevated utrophin expression levels [91]. Calcineurin thus acts both transcriptionally and post-transcriptionally to sustain utrophin expression. Despite the recuperative merits of calcineurin activation, DMD is characterised by depressed calcineurin and NFAT levels. Blood analyses of patients with DMD demonstrated significantly reduced calcineurin activity when compared to female carriers of the mutated DMD gene or healthy controls [92]. Calcineurin compensation can hence be improved in DMD.

The calcineurin pathway mobilises an innate recovery response pathway for counteracting multiple aspects of the dystrophic pathology. Through its coordinated promotion of oxidative muscle remodelling, maintenance of sarcolemmal integrity, suppression of inflammatory damage and enhancement of utrophin expression, calcineurin signalling has emerged as a promising therapeutic target for the treatment of X-linked muscular dystrophies. Developing non-invasive methods of restoring TRPC1-mediated Ca^2+^ entry may provide a realisable means of engaging this protective response. Other than mechanical forces, TRPC1 is also responsive to diverse biophysical stimuli. Non-invasive biophysical stimulation thus may provide a means of commandeering this important signalling pathway without exacerbating mechanical damage of fragile dystrophic muscle, despite the absence of dystrophin.

## 5. PEMFs as a Non-Invasive Strategy to Restore Calcineurin Signalling

Given that TRPC1 is responsive to diverse biophysical stimuli [46,57], it may remain therapeutically accessible via these means in cases of dystrophin deficiency. A TRPC1–calcineurin signalling axis was previously shown to be activated by pulsed electromagnetic fields (PEMFs). In accordance with noted calcineurin activation, brief exposure (10 min) to low-energy PEMFs (1 mT @ 15 Hz) was shown to promote oxidative muscle development in vivo and in vitro [56,72]. In this capacity, PEMFs act according to the principles of mitohormesis, whereby magnetic field exposure stimulates a TRPC1–calcineurin signalling axis that modulates mitochondrial adaptation via the PGC-1α and Nrf2 transcriptional cascades that, in turn, favour the expression of oxidative muscle [46,56,57]. Moreover, TRPC1 was shown to be necessary and sufficient for magnetic mitohormetic responses [93]. Accordingly, genetic or pharmacological interruption of TRPC1 signalling abolished these responses, highlighting the central role of this mechanosensitive/magnetosensitive channel in mediating muscle adaptation [56]. Finally, shielding myoblasts from all ambient magnetic fields reverted the same genetic and epigenetic oxidative pathways stimulated by supplemental magnetic fields, including TRPC1 expression. Isolation from magnetic fields also mechanistically mimicked pharmacological blockage of TRPC1 [56], indicating that magnetic fields are a true myogenic developmental imperative acting via TRPC1 [65]. TRPC1 serves as an integrator of diverse biophysical stimuli including mechanical forces, light and magnetic fields [42,57]. Those seeking greater mechanistic details on the process of magnetoreception are referred to the following source [65]. These findings establish the existence of a functional TRPC1–calcineurin signalling axis that is responsive to external magnetic stimulation that may remain therapeutically accessible in DMD.

TRPC1-mediated Ca^2+^ entry activates calcineurin and NFAT signalling [43,56,94], while calcineurin–NFAT activity sustains TRPC1 expression [43,56,95], establishing a positive regulatory network that supports oxidative muscle development by cooperatively stimulating mitochondrial biogenesis via PGC-1α [46,56]. A common feature of both PEMF stimulation and mechanical stimulation is their ability to engage the calcineurin–NFAT pathway as well as TRPC1 activation [27,43,57,58,72]. On the other hand, developmentally appropriate mechanotransduction is altered in the absence of dystrophin [8,14]. Therefore, a remaining question is whether TRPC1 can be activated by magnetic fields in the absence of dystrophin and cytoskeletal interaction. To address this caveat, we have previously shown that cell-derived vesicles with minimal cytoskeletal interaction, but containing only TRPC1 and TRPA1, could be loaded with Ca^2+^ by brief magnetic exposure, even while being mechanically unloaded in suspension [93], initially suggesting that TRPC1 can be activated by magnetic fields without cytoskeletal engagement. In terms of Ca^2+^ entry, persistent and repetitive activation of TRPC1 was shown to preferentially promote oxidative muscle development [96,97], aligning with data showing that sustained intracellular Ca^2+^ levels preferentially activate the NFATc1 isoform to promote oxidative muscle, whereas transient and high-amplitude Ca^2+^ elevations preferentially activate NFATc2 to promote glycolytic muscle development [71]. Provocatively, brief exposure to PEMFs (10 min) at 15 Hz has been shown to also be capable of activating TRPC1-mediated Ca^2+^ entry and promote oxidative muscle development in vitro [56] and in vivo [72], presumably without altering the localisation of TRPC1 within muscle. Collectively, these findings suggest that PEMF therapy can modulate TRPC1-mediated calcium signalling to re-engage the protective calcineurin-dependent transcriptional program and restore aspects of this adaptive signalling network that become compromised during X-linked muscular dystrophy disease progression.

The mechanistic framework outlined above suggests that restoring adaptive calcineurin signalling may represent a potential therapeutic avenue for ameliorating the muscle pathology of DMD. While current treatment strategies target either the primary genetic defect or downstream symptoms, the possibility of activating endogenous protective signalling cascades in fortifying muscle oxidative adaptation, regeneration and function may offer synergistic value. Achieving this would establish a stronger foundation, enabling other interventions to deliver greater therapeutic benefits. PEMF therapy emerges as a notable candidate because of its non-invasive manner of activating calcineurin signalling. A key advantage of PEMF exposure is that it does not impose additional structural stress on muscle, as it does not induce muscle contraction. This notable distinction will be especially relevant in DMD, where excessive mechanical strain exacerbates the damage to fragile dystrophin-deficient muscle fibres. The restoration of the TRPC1–calcineurin signalling in DMD muscle by PEMF exposure will confer benefits beyond just oxidative muscle remodelling and mitochondrial adaptation, which are associated with better disease prognosis. Calcineurin functions as both a transcriptional regulator of utrophin expression through NFAT-dependent activation of the utrophin A promoter and as a post-transcriptional regulator through enhanced stabilisation of utrophin A mRNA [84,91]. Consequently, by re-engaging the upstream TRPC1–calcineurin–NFAT–PGC-1α signalling axis, PEMF exposure may augment utrophin expression. Utrophin upregulation, in turn, may promote the assembly of additional UGCs at the sarcolemma, further helping to restore membrane-associated structural and signalling deficiencies resulting from the loss of dystrophin [86]. Given that utrophin is capable of functionally compensating for the loss of dystrophin in DMD [81], restoration of the calcineurin signalling pathway in response to PEMF therapy may reduce muscle pathology, regardless of the underlying mutation of the dystrophin gene and without mechanical stress.

Evidence also suggests that activating TRPC1 would not inadvertently recruit the TRPC3-induced deteriorative sequalae. It has been previously shown that TRPC1, per se, is necessary and sufficient for magnetoreception to be manifested [93] and that TRPC1 and TRPC3 are oppositely regulated in response to TRPC1 activation by PEMF exposure [56]. Furthermore, co-expressing TRPC1 with TRPC3 suppresses Ca^2+^ permeation through TRPC3-containing heteromultimeric channels without influencing TRPC3 transcript or protein levels [69,98]. These diverse pieces of evidence suggest that TRPC1 and TRPC3 activation do act additively, but in a cross-modulatory manner.

Finally, given that a fast-to-slow (glycolytic-to-oxidative) muscle fibre transition is a natural adaptation to exercise training that is evolutionarily intended to improve functional capacity, it is unlikely that our magnetic intervention, capable of inducing the same muscle fibre transition, is physically limiting. In fact, in can be argued that, in the context of DMD, any improvement in muscle expression, particularly oxidative, is a functional improvement.

## 6. PEMF Therapy May Ameliorate DMD Inflammatory Status

A TRPC1–mitochondrial signalling axis was identified, which promotes muscle adaptive survival and oxidative status as a consequence of PGC-1α transcriptional activation [56]. The Ca^2+^ overload caused by the absence of dystrophin instead disrupts muscle mitochondrial function and PGC-1α expression [99], which interferes with the adaptation of muscle towards a more oxidative phenotype. In response to PGC-1α activation, oxidative muscle releases into the systemic circulation a myriad of anti-inflammatory and metabolism stabilising cytokines [100] and is the underlying reason why aerobic exercise attenuates systemic inflammation [46]. As oxidative muscle establishes the inflammatory status of the entire organism, the disruption of this TRPC1–mitochondrial signalling axis would explain the metabolic disruption observed in DMD [18]. Accordingly, it has been shown that the functional expression of the DGC, and associated proteins such as α_1_-syntrophin and TRPC1 [20,21], correlates best with the oxidative phenotype [101]. A magnetic signal was initially optimised to specifically activate this TRPC1–mitochondrial signalling axis [56]. PEMF exposure applied at an amplitude of 1.5 mT at 15 Hz for 10 min was shown to promote mitochondrial respiration, mitochondrial survival adaptations, and oxidative muscle development in vitro [56] and in vivo [72], downstream of PGC-1α upregulation. This mitochondrial adaptive process, sometimes referred to as Magnetic Mitohormesis, is associated with increased muscular mitochondrial biogenesis, enhanced mitochondrial anti-oxidant defences, and reduced apoptosis [56]. Further optimisation of this PEMF signature for improved mitohormetic outcomes may still be experimentally achievable but remains to be shown. The glycolytic to oxidative shift produced by PEMF-mediated TRPC1 activation should hence ameliorate the high inflammatory status that characterises and aggravates DMD [18].

## 7. Synopsis

Collectively, the reported findings suggest that non-invasive PEMF therapy may serve as a catalytic surrogate for mechanical stimulation with reference to calcineurin activation, but not by acting as a vicarious form of mechanical stimulation per se, as this form of low-energy magnetic field exposure does not cause muscle contraction. PEMF therapy has been shown to activate the same adaptive TRPC1–calcineurin signalling axis as mechanical stimuli governing oxidative muscle remodelling and enhanced mitochondrial function. The absence of dystrophin in DMD, however, structurally and functionally disrupts this TRPC1–calcineurin signalling axis and impedes its appropriate activation by mechanical forces, as well as makes muscle unable to sustain the stress of normal contractile forces. Paradoxically, the activation of calcineurin in DMD skeletal muscle would be capable of mobilising a compensatory response pathway that induces the expression of a dystrophin homologue, known as utrophin, that can functionally substitute for the absence of dystrophin at the muscle surface, but is unable to do so due to the absence of dystrophin and disruption of TRPC1 signalling. The advantages of activating the calcineurin pathway in DMD have been clear for over two decades. The unmet need was a manner to appropriately activate the TRPC1–calcineurin axis in the absence of dystrophin and without augmenting mechanical stress. An option may now be available in the form of low energy PEMF-based therapies that may help to restore calcineurin signalling competence and improve the structural resilience of dystrophic muscle via this compensatory pathway. Mechanistically, PEMF-based therapies hold a yet-unexplored potential to activate endogenous calcineurin-dependent compensatory mechanisms. They achieve this by first restoring TRPC1-mediated calcium signalling without mechanical input; second, by promoting the oxidative muscle phenotype, which exhibits the greatest resistance to muscle damage in DMD; and lastly, by inducing the expression of utrophin to compensate for the absence of dystrophin at the DGC. Available data thus provide sufficient plausible mechanistic rationale to warrant future investigation of PEMF-based therapies as adjuvants to current DMD interventions. The prospects are good but remain to be clinically realised.

## Data Availability

No new data were created or analysed in this study. Data sharing is not applicable to this article.
